# Diagnostic accuracy of nasal nitric oxide for establishing diagnosis of primary ciliary dyskinesia: a meta-analysis

**DOI:** 10.1186/s12890-015-0147-3

**Published:** 2015-12-03

**Authors:** Panayiotis Kouis, Stefania I. Papatheodorou, Panayiotis K. Yiallouros

**Affiliations:** Cyprus International Institute for Environmental & Public Health in Association with Harvard School of Public Health, Cyprus University of Technology, 95 Irenes Street, 3041 Limassol, Cyprus; Department of Pediatrics, Hospital “Archbishop Makarios III”, Nicosia, Cyprus

**Keywords:** Primary Ciliary Dyskinesia, Nitric oxide, Kartagener syndrome, Diagnosis

## Abstract

**Background:**

To date, diagnosis of Primary Ciliary Dyskinesia (PCD) remains difficult and challenging. We systematically evaluated the diagnostic performance of nasal Nitric Oxide (nNO) measurement for the detection of PCD, using either velum-closure (VC) or non-velum-closure (non-VC) techniques.

**Methods:**

All major electronic databases were searched from inception until March 2015 using appropriate terms. The sensitivity and specificity of nNO measurement was calculated in PCD patients diagnosed by transmission electron microscopy, high speed video-microscopy or genetic testing. Summary receiver operating characteristic (HSROC) curves were drawn using the parameters of the fitted models.

**Results:**

Twelve studies provided data for 13 different populations, including nine case–control (*n* = 793) and four prospective cohorts (*n* = 392). The overall sensitivity of nNO measured by VC techniques was 0.95 (95 % CI 0.91–0.97), while specificity was 0.94 (95 % CI 0.88–0.97). The positive likelihood ratio (LR+) of the test was 15.8 (95 % CI 8.1–30.6), whereas the negative likelihood ratio (LR-) was 0.06 (95 % CI 0.04–0.09). For non-VC techniques, the overall sensitivity of nNO measurement was 0.93 (95 % CI 0.89–0.96) whereas specificity was 0.95 (95 % CI 0.82–0.99). The LR+ of the test was 18.5 (95 % CI 4.6–73.8) whereas the LR- was 0.07 (95 % CI 0.04–0.12).

**Conclusions:**

Diagnostic accuracy of nNO measurement both with VC and non-VC maneuvers is high and can be effectively employed in the clinical setting to detect PCD even in young children, thus potentiating early diagnosis. Measurement of nNO merits to be part of a revised diagnostic algorithm with the most efficacious combination of tests to achieve PCD diagnosis.

## Background

Primary ciliary dyskinesia (PCD) is a rare, hereditary disorder characterized by impaired mucociliary clearance [1]. Apart from situs inversus in ~50 % of the cases, the main manifestations of the disease are not specific. Nevertheless, the associated recurrent sinopulmonary infections eventually lead to severe chronic lung disease and development of bronchiectasis [2, 3].

While some centers began using targeted genetic testing [4], the diagnosis of PCD in the majority of centers currently relies on an array of different sophisticated tests namely the High Speed Video Microscopy (HSVM) for ciliary motility assessment [5], Transmission Electron Microscopy (TEM) for the examination of cilia ultrastructure [6] and nasal nitric oxide (nNO) measurement [7]. The diversity of the employed diagnostic tests reflects the lack of a golden diagnostic standard and the weaknesses and inaccuracies that characterize each of these tests. In particular, TEM examination of ciliary axonemes exhibits normal ultrastructure in confirmed patients with biallelic mutations in certain disease-causing genes such as DNAH11 [8], while the motility patterns observed by HSVM vary widely depending on the implicated genetic variant [9, 10].

Nasal nitric oxide (nNO) is abnormally low in PCD patients [11] and it has been part of the diagnostic work-up in many PCD centers [12]. Current American Thoracic Society/European Respiratory Society (ATS/ERS) guidelines for nNO measurements recommend air aspiration via a nasal probe while the subject exhales through the mouth against resistance in order to maintain velum closure. Alternative techniques to maintain velum closure such as breath hold or pursed- lip breathing via the mouth are also acceptable [13]. However, velum closure requires cooperation and this precludes the performance of these techniques in young children. Few reports have investigated the discriminative ability of nNO measurements with the velum open as in the case of tidal breathing [3, 14] with encouraging findings for the usefulness of this technique in screening for PCD in younger children and adults unable to perform velum closure.

In view of the above specific restrictions and weaknesses, for the clinicians and the patients it remains of key importance to appraise the potential diagnostic value of each of the available diagnostic tests for PCD, in order to find its place in the armamentarium for elicitation of the diagnosis of the disease. A recent systematic review and meta-analysis summarized the published evidence on the measurement of nNO in PCD and reported on the mean difference of nNO production values obtained during velum closure techniques in PCD patients versus healthy controls (231 nL/min, 95 % CI: 193.3–268.9) and cystic fibrosis patients (114.1 nL/min, 95 % CI: 101.5–126.8) [15]. However, that report did not perform a meta-analysis on the diagnostic accuracy of nNO measurements in order to provide synthesized data on the potential diagnostic value this test may have in future algorithms for PCD diagnosis, which would be particularly informative in clinical decision making. The aim of this study was to systematically evaluate the diagnostic performance of nNO measurement as obtained either with a velum-closure or a non-velum-closure technique in screening for PCD so as to provide appropriate summary estimates of diagnostic accuracy with each breathing technique and demonstrate the summary trade-off between sensitivity and specificity across the included studies.

## Methods

### Search strategy and selection criteria

The electronic databases PubMed, SCOPUS, Cochrane Database of Systematic Reviews and Google Scholar were searched from inception until March 2015 using the keywords: ‘nasal nitric oxide’, “nNO”, “nasal NO”, “Primary Ciliary Dyskinesia”, “PCD”, “lung”, “pulmonary”, “pulm*”, “cilia” either in the title or the abstract or using MeSH terms. The references of eligible studies were further examined for possible missing articles. We included studies which were identified after two reviewers (PK, SIP) independently screened the title and abstract of the obtained search results. Final selection was based on full text evaluation. Any disagreements were resolved by discussion and in case of discrepancy, by a third researcher (PKY). As this study is based on a systematic review of the previously published literature, an ethical approval was not obtained, since there is no potential of participant identification and ethical approval and consent was already obtained at the individual study level. The guidelines of the Preferred Reporting Items for Systematic Reviews and Meta-Analyses (PRISMA) were followed.

The validity of each primary study was assessed using the Quality Assessment of Diagnostic Accuracy Studies −2 (QUADAS-2) tool [16], that evaluates the risk of bias and applicability of diagnostic accuracy studies. It consists of four key domains: patient selection, index test, reference standard, flow and timing. Each is assessed in terms of risk of bias and the first three in terms of issues regarding applicability.

Studies were considered eligible if they provided data on the sensitivity and specificity of nNO for the diagnosis of PCD in order to construct a 2 × 2 table for each study calculating true positives (TP), false negatives (FN), false positives (FP) and true negatives (FN) for the presence or not of PCD according to nNO values set as a cut-off in each study. In some studies, the numbers were not provided per se but it was possible to extract them from other manuscript data sources. In case of incomplete information, we contacted the authors of the primary studies. Studies that reported only mean values of nNO were not included in our analyses as they did not provide data for computing summary diagnostic accuracy estimates (sensitivity, specificity, positive and negative likelihood ratio). Disease status in each selected study was required to have been confirmed by TEM and/or HSVM or genetic testing. Additional information on NO analyzer type, flow rate and breathing maneuver was also collected and used in data synthesis. Studies that did not report the equipment and flow rate used were not considered eligible as well as studies that may have used flow rate outside of the ATS/ERS recommended range (0.25–3 L/min) [13]. Cut-off values for the nNO test, were usually reported in parts per billion (ppb) and were transformed to NO production rate units (nl/min), using the conversion formula concentration (ppb) × sampling rate (L/min) as used previously [7], in order to account for the used different flow rates. Breathing maneuvers such as breath hold (BH) and exhalation against resistance (ER) were categorized as velum closure (VC) techniques and in case of both maneuvers performed by the study subjects; only results for the ER maneuver were included as the most validated technique according to ATS/ERS guidelines [13]. For studies employing the non-velum closure (non-VC) technique, only results of nNO measurements that were performed during tidal breathing (TB) with mouth open were included in the meta-analysis.

### Data extraction

The name of author, study design, publication year, country of origin, study population sample size, age distribution of study population subgroups, nNO cut-off levels, information on the measurement method and the test(s) used for the diagnosis of PCD were recorded for each study. Data on the values of TP, TN, FP, FN were extracted independently by two reviewers (PK, SIP). A third investigator (PKY) settled any discrepancies and consensus was reached for all data.

### Analysis

A bivariate model was used to calculate estimates of overall sensitivity and overall specificity. We fitted a two-level mixed logistic regression model conditional on the sensitivity and the specificity of each study and a bivariate normal model for the sensitivity and specificity between studies [17]. This method combines information from multiple thresholds and the output is expressed as a hierarchical summary receiver operator curve (HSROC). The HSROC describes the relationship between sensitivity and specificity derived from the individual receiver operator curves (ROC) of each study. Following this method, it describes the ‘average’ relationship between a continuous cut-off value and discriminatory ability in the ‘average’ population. This maximizes the amount of information used in the evidence synthesis and better represents the available data. The advantage of this method is that it allows clinicians to estimate how changing thresholds will alter the diagnostic utility of the test under study. All calculations are performed using STATA (Version 12, StataCorp, College Station, Texas) with the commands metandi and metandi plots for analyses of four studies and above [18].

We also reported the summary likelihood ratios across all studies. These measures also combine in their calculation both sensitivity and specificity. Positive likelihood ratio (LR+) is the ratio of sensitivity/(1-specificity), whereas negative likelihood ratio (LR-) is defined as the ratio of (1-sensitivity)/specificity. When there is absolutely no discriminating ability for a diagnostic test, both ratios are equal to 1. The discriminating ability is better with higher LR+ and lower LR-. A good diagnostic test has typically LR+ greater than 5.0 and LR- less than 0.2 [19].

VC and non-VC measurements were analyzed separately and this allowed us to arrive at estimates on overall sensitivity, specificity and likelihood ratios for nNO depending on VC status. We also performed a sensitivity analysis including only studies in which PCD status was defined by TEM and at least one more diagnostic test, with the rationale to examine whether the diagnostic accuracy of nNO measurement differs with the inclusion of a more representative spectrum of PCD population. Measurements of nNO were compared to PCD diagnosis obtained through a combination of tests which included TEM and HSVM or DNA testing.

## Results

### Eligible studies

**Fig. 1:**
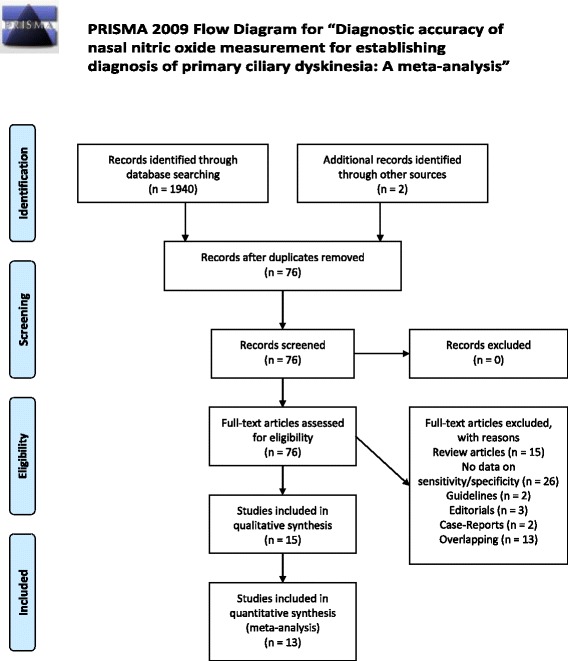
PRISMA diagram. PRISMA diagram for the search strategy and selected studies. *From:* Moher D, Liberati A, Tetzlaff J, Altman DG, The PRISMA Group (2009). Preferred Reporting Items for Systematic Reviews and Meta-Analyses: The PRISMA Statement. PLoS Med 6(6):e1000097. doi:10.1371/journal.pmed1000097. For more information, visit www.prisma-statement.org

Of the 1940 items retrieved through online search, 1866 were excluded based on the title and abstract and the remaining 74 were downloaded for detailed, full text assessment. Two additional studies were identified through references screening and were also evaluated. Studies with overlapping populations were cross-checked and final selection was based on the largest number of participating PCD patients. In summary, 26 studies did not provide data on sensitivity and specificity, 13 items involved overlapping populations, 15 items were review papers while the remaining items that were excluded were case reports (2), editorials (3) and guidelines papers (2) (Fig. 1). Of the total 76 studies assessed in detail, 15 provided enough data for the construction of a 2×2 table. Among these, two studies did not report type of NO analyzer and flow rate and despite our effort to obtain this information after contacting the authors, this was not feasible and they were excluded from the analysis [20, 21]. Finally, quantitative synthesis included data on 13 different populations from 12 studies (Marthin et al. included data on more than one population) and two separate analyses were carried out, based on the employed breathing maneuver.

### Study characteristics

Descriptive characteristics of the included studies are presented in Table 1. From 12 studies, 325 PCD patients and 711 non-PCD subjects were included in the meta-analysis for the diagnostic performance of VC nNO testing. In the case of non-VC nNO testing, 210 PCD patients and 471 non-PCD subjects from seven studies were included. The majority of the studies were performed in Western Europe and only two in North America. Four studies evaluated the diagnostic efficacy of nNO in cohorts of referred suspect patients for PCD testing [7, 22–24] whereas the rest of the studies had a case–control design. Controls were non-PCD subjects, either healthy subjects only [22, 25–28] or healthy subjects and patients with other respiratory diseases [14, 29–31].

**Table 1:** Characteristics of the included studies

#	Author/Study	Country	Study Design	Study Population^a^	Age range (yrs) (Mean, range/SD)	Analyzer	Flow rate (L/min)	Measurement Method^b^	Cut – off (nL/min)	Inclusion criteria/ Diagnosis
1	Narang I (2002) [26]	United Kingdom	Case - Control	31 PCD 53 HC	PCD: 11.0 (5.5–17.3) HC: 10.7 (5.5–19.0)	LR 2000	0.25	BH	62.5	HSVM and TEM
2	Corbelli R (2004) [23]	Switzerland	Prospective Cohort	17 PCD 17 non PCD (BE,B)	All: 11.4 (1.2)	CLD88sp	1.20	BH	126	TEM
3	Piacentini G (2008) [25]	Italy	Case - Control	10 PCD 27 HC	PCD: 17 (−) HC: 7 (−)	NIOX Flex	0.30	BH	21.3	TEM
4	Mateos Coral D (2011) [31]	Canada	Case – Control (with longitudinal follow-up in a subsample)	20 PCD 65 non PCD (CF,BE,HC)	PCD: 11.4 (3.5) HC: 11.0 (3.7) CF: 11.0 (3.4) BE: 10.9(3.3)	CLD88sp	0.33	ER & TB	ER: 58.5 TB: 37.1	TEM
5	Marthin JK (2011) [22] Substudy 3	Denmark	Prospective Cohort	20 PCD 97 non PCD	All: 6.9 (0.0–62.4)^c^	NIOX Flex	0.30	BH & TB	BH: 52.5 TB: 47.4	HSVM and TEM
6	Marthin JK (2011) [22] Substudy 2	Denmark	Case - Control	59 PCD 57 HC	PCD: 17.4 (3.6–65.8)^c^ Non PCD:29.5 (3.1–63.6)^c^	NIOX Flex	0.30	BH & TB	BH: 52.5 TB: 47.4	HSVM and/or TEM
7	Leigh M (2013) [7]	United States	Prospective Cohort	71 PCD 84 non-PCD	PCD: 23.3(18) Non PCD: 31.8 (22.3)	Sievers 280i CLD88sp NIOX Flex	0.50 0.33 0.30	ER	76.9	TEM and DNA
8	Boon M (2014) [30]	Belgium	Case - Control	38 PCD 188 non PCD (HC, CF, Asthma, HID)	PCD: 14.3 (8.8–18.1)^c^ HC: 14.9 (10.8–20.4)^c^ CF: 14.0 (9.2–17.9)^c^ Asthma: 12.1 (9.8–16.5)^c^ HID: 10.7 (8.2–15.6)^c^	CLD88sp	0.30	ER & TB	ER: 90 TB: 60	HSVM and TEM (and culture)
9	Harris A (2014) [14]	United Kingdom	Case - Control	13 PCD 37 non PCD (HC,CF, CSLD)	PCD: 23 (5–71) HC: 31 (8–65) CF: 15(6–29) CSLD: 36 (8–79)	NIOX Flex NIOX MINO	0.30	BH & TB	BH: 38 TB: 30	HSVM and TEM (and culture for some)
10	Montella S (2012) [27]	Italy	Case - Control	23 PCD 23 HC	PCD: 15.8 (4.6–32.8)^c^ HC: 15.7 (4.3–32.1)^c^	NIOX MINO	0.30	TB	17.4	HSVM and TEM
11	Santamaria F (2008) [28]	Italy	Case - Control	14 PCD 14 HC	PCD: 15 (7–27) HC:16 (7–27)	NIOX Flex	0.28	BH	7.2	TEM
12	Moreno Caldo A (2010) [29]	Spain	Case Control	9 PCD 112 non PCD (HC, CF, Asthma,BE)	PCD: − (7–14) HC: − (−) CF: − (6–14) Asthma: (6–17) BE: − (6–14)	LR2000	0.25	BH	28	TEM
13	Beydon M 2015 [24]	France	Prospective Cohort	49 PCD 37 non-PCD	PCD: 11.4 (7,13.9)^d^ Non PCD: 7.9 (4.9,11.6)^d^	NIOX Flex Endono 8000	0.30	BH/ER TB	BH/ER: 82.2 TB: 39.9	HSVM, TEM and/or DNA

PCD: Primary Ciliary Dyskinesia, HC: Healthy Controls, B: Bronchitis, CF: Cystic Fibrosis, BE: non CF non PCD Bronchiectasis, CSLD: Chronic Suppurative Lung Disease, HID: Humoral Immunodeficiency Disorders, TEM: Transmission Electron Microscopy, HSVM: High Speed Video Microscopy, DNA: Genetic testing

^a^Study population refers to subgroups that comparisons (sensitivity, specificity, PPV, NPV) were reported for in the original articles

^b^Measurements methods taken into account for the meta-analysis, BH: Breath Hold, ER: Exhalation against Resistance, TB: Tidal Breathing

^c^Median (range)

^d^Median (IQR)

**Fig. 2:**
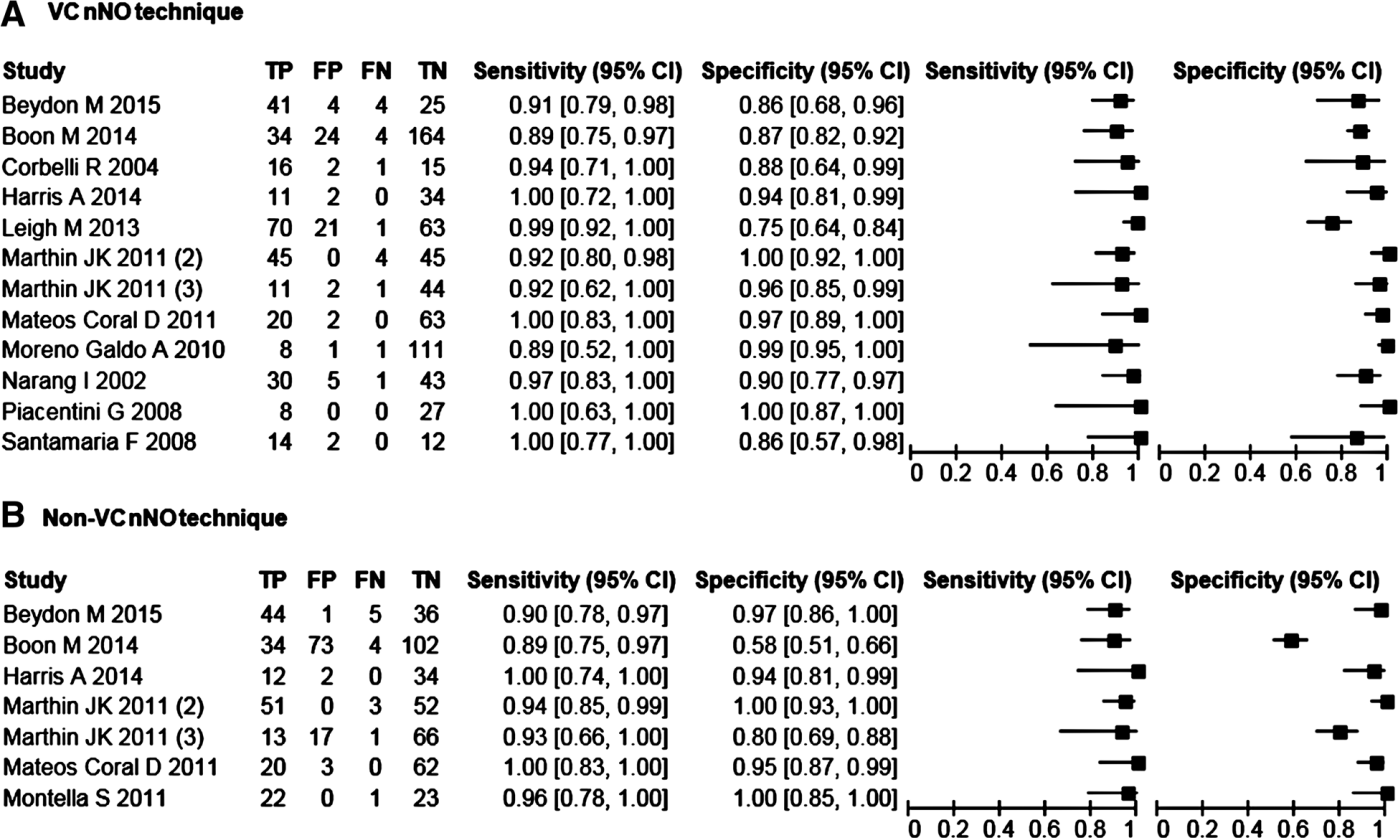
Forest plots for sensitivity and specificity. Forest plot of sensitivity and specificity of nNO for detecting PCD with the 95 % CI for each population of the included studies. **a** Forest plot for studies employing a VC breathing technique and **b** Forest plot for studies employing a non-VC breathing technique

The number of PCD patients (range: 9–59) and controls (range: 14–188) per case–control study varied widely. All case–control studies confirmed PCD status by TEM findings while in 55 % of them HSVM was also performed. Of the four prospective studies, Beydon et al. used a combination of TEM, HSVM and genetic testing to confirm PCD diagnosis [24] while Marthin et al. used TEM and HSVM in their cohort of consecutive referrals [22]. Leigh et al. confirmed PCD via a combination of ultrastructure assessment and genetic testing [7] while the smallest cohort study confirmed PCD only via ultrastructural assessment [23]. The sensitivity and specificity of each included study with VC and non-VC technique are shown in Fig. 2.

### Quality assessment

Reporting of the meta-analysis is based on PRISMA guidelines [32]. Based on the QUADAS-2 tool, the quality assessment of the primary studies is shown in Table 2. In general, the analyzed studies had overall reasonably good methodology and this offers relative reassurance that results have not been substantially influenced from bias.

**Table 2:** QUADAS-2 Quality Assessment results

Study	Risk of bias				Applicability concerns		
	Patient selection	Index test	Reference Standard	Flow and timing	Patient selection	Index test	Reference standard
Boon 2014 [30]	L	U	L	L	L	L	L
Mateos Coral 2011 [31]	U	L	L	L	U	L	L
Piacentini 2008 [25]	U	L	L	L	U	L	L
Santamaria 2008 [28]	L	U	L	L	L	L	L
Montela 2012 [27]	U	L	U	L	L	L	L
Corbelli 2004 [23]	L	U	L	L	L	L	L
Narang 2002 [26]	L	L	L	L	L	L	L
Harris 2014 [14]	L	U	L	L	L	L	L
Leigh 2013 [7]	L	L	L	L	L	L	L
Marthin 2011 [22]	L	U	L	L	L	L	L
Moreno Galdo 2010 [29]	U	U	L	L	U	L	L
Beydon M 2015 [25]	L	L	L	U	L	L	L

QUADAS 2 consists of four key domains covering patient selection, index test, reference standard and flow of patients through the study and timing of the index test and reference standard (“flow and timing”). Each domain is assessed in terms of the risk of bias and the first three are also assessed in terms of concerns regarding applicability

U: Unknown, L: Low, H: High

### Data synthesis

**Fig. 3:**
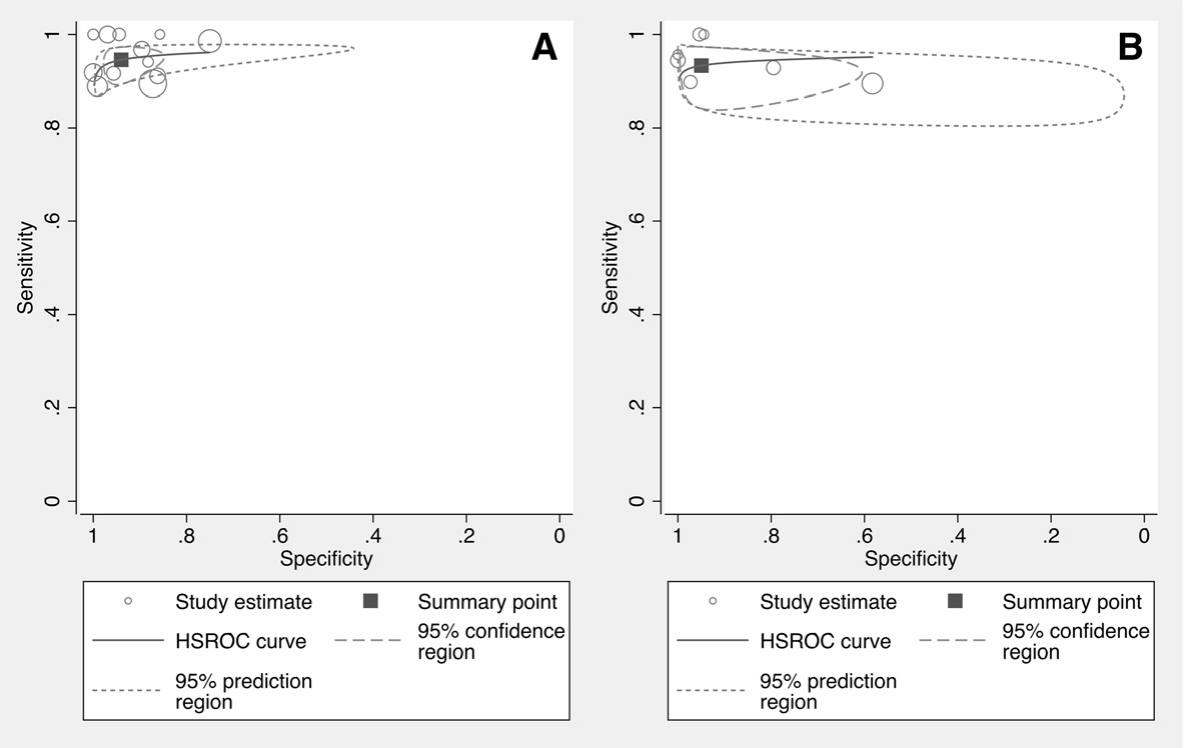
VC and non-VC HSROC curves. HSROC curves for the included studies. **a** HSROC curve for studies employing a VC breathing technique and **b** HSROC curve for studies employing a non-VC breathing technique

The overall sensitivity of abnormal (low) nNO measured by VC techniques for all the included studies was 0.95 (95 % CI 0.91–0.97), while the specificity was 0.94 (95 % CI 0.88–0.97). The LR+ of the test was 15.8 (95 % CI 8.1–30.6), whereas the LR- was 0.06 (95 % CI 0.04–0.09). The HSROC curve is shown in Fig. 3.

For the non-VC techniques the overall sensitivity of nNO to detect PCD was 0.93 (95 % CI 0.89–0.96) whereas the specificity was 0.95 (95 % CI 0.82–0.99). The LR+ of the test was 18.5 (95 % CI 4.6–73.8) whereas the LR- was 0.07 (95 % CI 0.04–0.12). The HSROC curve is shown in Fig. 3.

When we performed a sensitivity analysis, to calculate the nNO diagnostic accuracy in studies that only included PCD populations diagnosed by more than one test (combination of TEM and HSVM or genetic testing) [7, 14, 22, 24, 26, 27, 30], the results did not change significantly. Similarly, after performing a post hoc sensitivity analysis, with the exclusion of studies that used the electrochemical devise NIOX MINO [14, 27], the resulting estimates of overall sensitivity and overall specificity for the non-VC maneuver do not significantly differ from the estimates of the main analysis.

## Discussion

In this meta-analysis we demonstrated that nNO measurement with VC techniques has overall sensitivity of 95 % and a specificity of 94 % whereas nNO measurement with the non-VC technique has comparable and very similar sensitivity (93 %) and specificity (95 %). We applied a different approach to the one employed in a previous report [15] and evaluated nNO diagnostic performance metrics by using all the available evidence in the literature. These summary estimates allow us to make comparisons between the various proposed and established diagnostic tests for PCD, which are essential for clinical decision making. We also provide a graphical representation of our results using the hierarchical summary receiver operating characteristic (HSROC) curve incorporating the different cut-offs between primary studies. The clinical utility of nNO measurement is underlined by the high LR+ (VC: 15.8, non-VC: 18.5) and low LR- (VC: 0.06, non-VC: 0.07), meaning that an abnormal (low) nNO leads to a steep increase in the post-test probability of PCD, compared to the pretest probability, while in the case of a normal nNO measurement the opposite is also true [19]. However, since the sensitivity and specificity of the test are not 100 %, in the presence of strong clinical suspicion for PCD [33], even in the case of a negative nNO test, a more detailed diagnostic work-up (HSVM, TEM, genetics) is indicated.

Current ATS/ERS recommendations for nNO include only VC maneuvers although recent evidence [14, 30, 31], that is supported by the results of this meta-analysis, highlights the discriminative ability of nNO during TB. TB is the only method available to obtain nNO measurements in young children (<5 years), which is particularly important as disease manifestations appear very early in life. Of course, the validity of nNO measurements in infants (<6 months) has been questioned, as nNO output in infancy is reduced due to the partial development of paranasal sinuses [25] where the majority of NO is produced [11] whereas the number of patients under 5 years which were evaluated in these studies [14, 22, 24, 27, 30, 31] is very small . The usefulness of nNO during TB has been demonstrated in the Danish cohort of 117 consecutive referrals with median age 6.9 years, where 83 % were able to perform TB versus 50 % for BH and 31 % for ER [22]. There is evidence that patients that have earlier diagnosis of this disease might have better clinical and functional outcomes [34, 35] and the application of this promising screening method in preschool children could not only lead to diagnosis at an earlier age but could also contribute towards the reduction of unnecessary cilia biopsies. Nevertheless, our results for the non-VC techniques should be interpreted with caution. Their low 95 % CI limit for specificity is at 82 % which suggests that a significant number of suspect PCD referrals is possible to give a falsely low nNO and prompt further diagnostic testing, thus increasing costs both to the healthcare system and the patient. Only seven studies were eligible for inclusion in the meta-analysis of non-VC maneuver, as opposed to 12 studies for the VC maneuver, and the low 95 % CI limit of the former may be due to the limited sample size. Furthermore, two of the non-VC studies used the NIOX MINO portable device which uses electrochemical analysis of NO as opposed to the better validated chemiluminescence method of the stationary devices (NIOX FLEX, Ecomedics CLD88). Nevertheless, we performed a post hoc analysis with the exclusion of these studies which did not influence the diagnostic accuracy of the non-VC maneuver and the quantitative synthesis includes data from all seven non-VC studies.

NIOX MINO is a simpler and cheaper tool for measuring nNO, and validation studies have already been published [14, 36, 37]. However, as NIOX MINO was designed for exhaled NO measurement in asthmatic individuals, issues relating to its accuracy [37] and repeatability [14] have been reported when used for nNO measurement in subjects referred for PCD evaluation. In addition, while the manufacturer recommends measurements of nNO with BH for at least 45 s, this is usually not possible by many patients and instead NIOX MINO is frequently used with the alternative TB maneuver regardless of patient’s age [14, 37]. These limitations question the suitability of NIOX MINO as a stand-alone diagnostic test and additional studies on the diagnostic accuracy of NIOX MINO measurements during TB are needed to confirm the validity of this method. However, the low cost and simple use potentiate the consideration of NIOX MINO as a promising first line screening test in a future diagnostic algorithm for PCD.

Currently, there is no universally accepted cutoff for abnormally low nNO. The included studies in this meta-analysis proposed a variety of cutoffs for nNO production by VC (7.2–126 nl/min) and non-VC (17.4–60 nl/min) techniques. This variability demonstrates the need for standardization of nNO measurements and agreement on cutoffs for the different breathing maneuvers. A recent, large, multicenter study has proposed a cutoff equal to 77 nl/min for VC [7], whereas the meta-analysis by Collins et al. reported that a cutoff of 75.2 nl/min would include 99.85 % of PCD patients performing VC maneuvers [15]. Regarding the non-VC maneuver however, no cutoff value has been proposed by a large enough study, thus additional studies are needed for the establishment of such cutoffs and further standardization of the technique.

Our study has some limitations. The main limitation is the heterogeneity and the weaknesses of the diagnostic standard that the published studies are employing for the definition of PCD status. As a result, the captured spectrum of the disease might not be totally representative of the true PCD population. TEM, which is the most commonly used test for PCD status definition in published studies, misses approximately 30 % of patients with PCD [38] and this should be taken into account in the assessment of the diagnostic efficacy of nNO. However, in the sensitivity analysis, when we included the studies that had employed more than one (in addition to TEM) diagnostic test to establish PCD diagnosis, our results did not change substantially thus providing relative certainty to the accuracy of nNO as a diagnostic test. There is considerable variation between individual studies in the number of cases, total sample size and cut-off values. However, the bivariate meta-analysis and HSROC curve analyses take explicitly this diversity into account and can accommodate studies with populations of different risks and different definition thresholds. Another issue for the synthesis of the data is that the majority of the included studies were diagnostic case–control studies. Empirical evidence has shown that case–control studies, as opposed to cohort studies, may overestimate the diagnostics Odds Ratio (DOR) [39]. Nevertheless, we think that the possibility of overestimation is limited, as the case–control studies included here were diagnostic studies designed to assess the test accuracy and not to provide evidence on associations between a risk factor and the disease [40]. Additionally, given the rarity of PCD, it is expected that the majority of studies will have a case–control design. Due to the same reason, both case–control and prospective cohort studies included relatively small numbers of subjects. However the synthesis of the included studies led to the inclusion of data for several hundreds of PCD and non-PCD subjects and allowed the use of the appropriate statistical models and provided the estimates we report. It should be underlined of course that these estimates apply provided that the ATS/ERS guidelines are followed for the performance of the test and the obtained values are compared to the normal values obtained from samples of healthy subjects in the respective populations.

## Conclusions

In summary, measurement of nNO, both with VC and non-VC maneuvers, has high overall diagnostic accuracy and provides a clinically significant diagnostic tool for large uninvestigated populations of suspect cases worldwide where access to TEM and HSVM is not easy. Furthermore, the high overall diagnostic accuracy of nNO calls for re-evaluation of the diagnostic accuracy of each of the available diagnostic tests for PCD (nNO, TEM and HSVM) with the aim to develop an algorithm with the most efficacious combination of tests to achieve PCD diagnosis.

## Abbreviations

ATS/ERS: American Thoracic Society/European Respiratory Society
BH: Breath hold
ER: Exhalation against resistance
FN: False negatives
FP: False positives
HSVM: High Speed Video Microscopy
nNO: nasal Nitric Oxide
PCD: Primary Ciliary Dyskinesia
ppb: parts per billion
TB: Tidal breathing
TEM: Transmission Electron Microscopy
TP: True positives
TN: True negatives
VC: Velum closure
